# A Simple Method for the Screening and Measurement of Phenols in *Dendrobium chrysotoxum* by a Miniature Mass Detector Using a Matrix Solid-Phase Dispersion Method

**DOI:** 10.1155/2019/6737632

**Published:** 2019-01-23

**Authors:** Hongcheng Liu, Duo Mu, Tao Lin, Qiwan Li

**Affiliations:** ^1^Supervision and Testing Center for Farm Product Quality, Ministry of Agriculture, Institute of Quality Standard and Testing Technology, Yunnan Academy of Agricultural Science, Kunming 650223, China; ^2^Department of Pharmaceutical Science, Kunming Medical University, Kunming 650500, China

## Abstract

The present study aims at building a miniature mass method for the simultaneous determination of 12 phenols including the subtypes of bibenzyl, phenanthrene, and fluorenone, which was used to evaluate the quality of *Dendrobium chrysotoxum*. Through the full scan mode, new compounds were elucidated. The new compounds were quantified by carrying out the analysis of the ratio of the standard solution areas to new compound areas versus analyte concentration. The limit of detection (LOD) and limit of quantification (LOQ) for phenols were 0.5 *µ*g/mL–1 *µ*g/mL and 1 *µ*g/mL–2 *µ*g/mL, respectively. Average recoveries of phenols were ranged from 83.2% to 97.5%. Reproducibility represented by the RSD percentage was from 2.3% to 8.7%. The average content of the four analytes, erianin, chrysotobibenzyl, confusarin, and moscatilin, were more than 200 mg/kg, and the content of bibenzyl compounds was found to be the highest in *Dendrobium chrysotoxum.* Among these bibenzyl compounds, erianin was determined as the typical chemical marker from *Dendrobium chrysotoxum*. The newly established UPLC with a miniature mass detector method was found to be an appropriate tool for the quality assessment of *Dendrobium chrysotoxum*.

## 1. Introduction

There are 74 species of *Dendrobium* reported in China [1]; however, only few *Dendrobium* species are edible. *Dendrobium chrysotoxum* is used in traditional or folk Chinese medicine in westsouth China, which can be differentiated from the others only by specialists. And its quality assessment depends mainly on morphology-based authentication, which might be practical in distinguishing different species with distinct features, but is not effective for evaluating the quality of same species from different ecological conditions or at different cultivation stages. In Chinese Pharmacopoeia of 2015, the quality of *Dendrobium chrysotoxum* is assessed by the determination of erianin with high-performance liquid chromatography (HPLC). HPLC has been widely utilized for the analysis of pharmaceutical compounds and medicinal herbs. There are many published methods for HPLC analysis of bibenzyl and phenanthrene in *Dendrobium* [2–6]. However, these standard compounds can be separated only in laboratory, so it is difficult to use this method in routine analysis.

To overcome such problems, liquid chromatography (LC) coupled with mass spectrometry has been used [7, 8]. It was disadvantageous to these methods, such as complicated operation and expensive instrument. A new technology of miniature mass has been developed for environment pollution and drug analysis [9]. The developments in the medical field are focused on the raw materials and active pharmaceutical ingredients [10–15]. To our knowledge, no research on the qualitative and quantitative analysis of phenols from *Dendrobium chrysotoxum* by a miniature mass detector (MMD) has been previously reported.

Previous studies on the analysis of phenols involved a simple dilution of the sample or one simple extraction procedure [16, 17]. Matrix solid-phase dispersion (MSPD) is a unique technique, which is especially suitable to the extraction of solid, semisolid, and/or highly viscous food and biological matrices, and achieves the isolation of target analytes by dispersing tissues onto a solid support, thus avoiding many difficulties encountered by employing the classical solid-phase extraction (SPE) approach. The main benefits of MSPD include flexibility, selectivity, and the possibility of minimizing extraction and cleanup steps, resulting in a drastic reduction in the analysis time and lower solvent consumption [18, 19].

Therefore, we developed and validated a miniature mass method for the simultaneous determination of 12 phenols including the subtypes of bibenzyl, phenanthrene, and fluorenone, which was used for subsequent analysis of their content in *Dendrobium chrysotoxum*. Through the full scan mode, new compounds were monitored and quantified based on the ratio of standard solution areas to new compounds. The combination of a fast, low-cost extraction procedure and a rapid detection method gives a reliable screening method that can be applied in the routine laboratory analysis for the determination of these phenols, reducing the cost of analyses and increasing the sample throughput.

## 2. Experimental

### 2.1. Chemicals

Gigantol, erianin, chrysotobibenzyl, tristin, coumarin, naringenin, apigenin, moscatin, and confusarin were of the highest purity (purity > 98%) were supplied by Zhong Ke Technology Co. Ltd. (Beijing, China). The structures of the compounds are shown in Table 1.

The stock solutions of the reference compounds were prepared by dissolving the compounds in methanol, and the working standard solutions were prepared daily from stock solutions by diluting the solution with the appropriate volume of the mobile phase. All solutions were stored in a refrigerator at −20°C. HPLC-grade acetonitrile and methanol were provided by Tedia Company Inc. (OH, USA). Water was purified using a Milli-Q system (Millipore, Bedford, USA). The other solvents, purchased from Shanghai Chemical Factory (Shanghai, China), were of analytical grade.

### 2.2. Sample Preparation

#### 2.2.1. Plant Material

Four fresh samples (three kilograms) of *Dendrobium chrysotoxum* were supplied by Professor Shouling Li from Ruili *Dendrobium* Field Genebank, Yunnan Province, China. The samples were dried at 50°C for one week and grounded to powder using a Waring (Hunan, China) HD100 blender at high speed (20,000 rpm).

#### 2.2.2. Matrix Solid-Phase Dispersion

A 500 mg portion of the dried sample was put into a 50 mL beaker, and 1 g of Florisil and 0.5 g of C18 were added. The mixture was then blended with a glass pestle until it become homogeneous, after which the samples were allowed to stand for 15 min.

The samples containing absorbent were introduced into the cartridge (6 mL volume capacity). The cartridge was washed with 10 mL hexane and discarded. Ten milliliter of methanol was added, and the elution was collected in a 10 mL graduated tube. A 2 *μ*L portion of the elution was analyzed by UPLC.

#### 2.2.3. UPLC-Miniature Mass Detector (MMD) Analysis

An Acquity UPLC™ System (Waters, Milford, MA, USA), with a binary solvent manager and a sample manager, combined with a QDa detector (MMD), was used for analyzing the phenolic fraction separation and identification of the phenolic acids. The column used was a Waters Acquity UPLC BEH C18 (100 mm × 2.1 mm × 1.7 *μ*). The column temperature was set at 30°C, and the injection volume was 5 *µ*L. The solvents used were acetic acid 0.1% in water (mobile phase A) and methanol (mobile phase B). The gradient was as follows: 0–1 min 90% (A) and 10% (B), 1–10 min 35% (A) and 65% (B), and 10–12 min 10% (A) and 90% (B).

The MMD condition is explained in Section 2.2.4.

#### 2.2.4. Newly Elucidated Compound Analysis

*(1) Full-Scan Spectra of Standards: MMD Conditions*. The ESI source conditions were as follows: source temperature was set at 350 °C. The full-scan mode was performed in the range of 50–400 Da, the scan time rate was 8 pin/sec, and the capillary voltage was set to 0.8 kV, while the cone voltage was at 15 V. The positive and negative modes were simultaneously recorded (Table 1).

*(2) Full-Scan Spectra of Sample: MMD Conditions*. The ESI source conditions were as follows: source temperature was set at 350 °C. The full-scan mode was performed in the range of 50–400 Da, the scan time rate was 8 pin/sec, and the capillary voltage was set to 0.8 kV, while the cone voltage was at 15 V. The positive and negative modes were simultaneously recorded.

*(3) Quantification Determination: MMD Conditions*. The ESI source conditions were as follows: source temperature was set at 350°C. The MMD mass spectrometer was operated in the selected ion mode (SIM), and the experimental conditions were the same as those described in Step 2. Newly elucidated compound quantification was built by carrying out an analysis on the ratio of the family standard solution areas to newly elucidate compound areas versus analyte concentration (see Table 2).

### 2.3. Method Validation

The calibration curve with matrix-matched standards of gigantol, erianin, chrysotobibenzyl, tristin, moscatin, confusarin, coumarin, naringenin, and apigenin was obtained. Calibration curves ranging from 2 to 100 *µ*g/mL were constructed from serial dilutions of the standard. Six concentrations for each of the standard solutions were injected in triplicate, and then the calibration curves were constructed by plotting the peak areas versus the concentration of the corresponding standard. The limits of detection (LOD) and the limits of quantification (LOQ) were estimated by means of the baseline noise method with a signal three and ten times higher than that of the baseline noise, respectively (see Table 3).

The accuracy and precision of the whole analytical procedure were evaluated by a fortified sample at 50 and 200 mg/kg in five replicates at each level.

## 3. Results and Discussion

### 3.1. Optimization of the MSPD Condition

In the present work, MSPD is used for extraction, and the plant sample is dispersed over deactivated mixture of C18 and Florisil. Various tests with other solid supports, such as neutral alumina and Florisil, were performed. The recoveries of coumarin were lower than 60%, and the recoveries of confusarin were not satisfied with C18.

**Figure 1:**
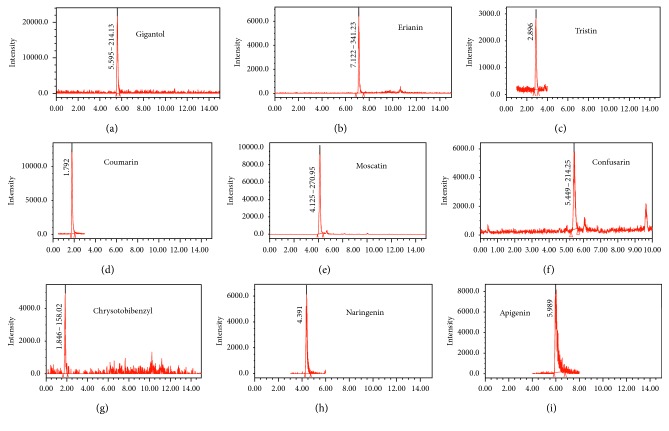
SIM chromatogram of the standard.

Different mixture rates of C18 and Florisil were applied to evaluate the capability of cleaning up from spiked samples. The recoveries of the method are shown in Figure 1. According to the above results, C18/Florisil (1 : 2) was selected as the final adsorbents used in the following studies.

In order to choose a proper elution for the retained phenols, various organic solvents were studied. When the samples and C18/Florisil were blended, which involved washing with hexane and then eluting with ethyl acetate, it was found that, with the exception of methanol, acetone and ethyl acetate could not elute phenols from the cartridge quantitatively. The *n*-hexane could not elute phenols from the cartridge, so *n*-hexane was selected as the clean solvent. Then, the phenols were eluted by methanol (10 mL).

### 3.2. Newly Elucidated Compound Confirmation and Quantification

All reported methods [1–3, 6], with *Dendrobium*, were required for the known compound analysis, but it was limited to obtain all needed standards by the laboratory.

Through the analytical strategy, we can screen the new compounds. First, the full-scan spectra of standards were used and the presence of the signal at the corresponding retention time (RT) was used to identify the target chemical or family of compounds. Second, the precursor ion and other mass spectra in the sample was recorded. If the characteristics of mass spectra were the same with standard and literature, the new compound was confirmed. Third, the new compound was quantified by the standard. Under the same condition, the standard was scanned for both in positive and in negative modes. For phenanthrene and coumarin, MMD in the positive mode can only give a powerful signal. Although MMD in the positive and negative mode can give a signal for bibenzyl and flavone, the flavone family in the negative mode has a more powerful signal than in the positive mode. However, the result of bibenzyl family was contrary, except for chrysotobibenzyl that lacks hydroxyl group, which did not give a signal in the negative mode. The characterise of bibenzyl was shown to format [M + Na]^+^ in the source which was more sensitive than the protonated adduct. Because of a nonvolatile element of sodium that can precipitate in the sampling cone at the laboratory in anyway, [M + Na]^+^ was thoroughly detected in the full-scan mode.

The selection of ions for the family of bibenzyl was based on the intensity of [M + Na]^+^ in the positive mode as well as the signal in the negative mode.

**Figure 2:**
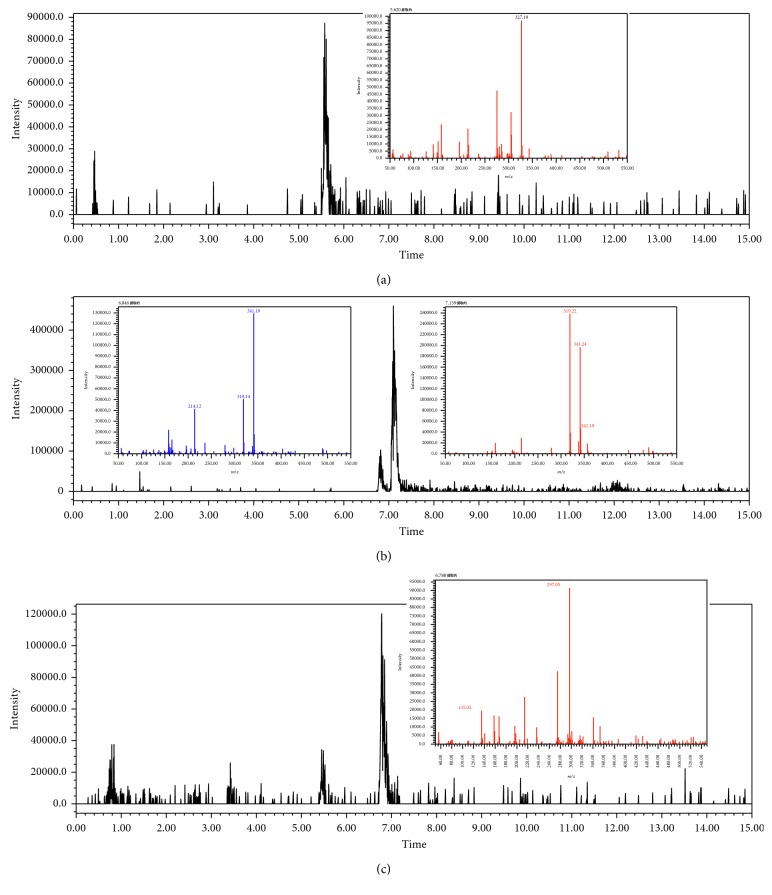
Full scan chromatogram and mass spectra of identified bibenzyl in sample (a) moscatilin; (b) chrysotoxin; (c) 3,4-dihydroxy-5,4'-dimethoxybibenzyl.

Figure 2 shows an overview of the full-scan spectra of the sample. For the peak of 5.62 min, the base peak was [M + Na]^+^ = 327 and [M + H]^+^ = 305 with relative abundant of 48%, and it existed with signals in the positive and negative modes. The result showed that it was a characteristic of bibenzyl. Through searching the literature reported in [1], the compound was found be moscatilin.

For the peak of 6.84 min, it was not matched with the retention time and mass spectra of erianin, the base peak was [M + Na]^+^ = 341 and [M + H]^+^ = 319 with relative abundant 48%, and it existed with signals in the positive and negative modes. Through searching the literature [1], the compound was found be chrysotoxin.

For the peak of 6.97 min, it was the same with gigantol, the base peak was [M + Na]^+^ = 297 and [M + H]^+^ = 275 with relative abundant of 50%, and it existed with signals in the positive and negative modes. The compound should be 3,4-dihydroxy-5,4′-dimethoxybibenzyl as per the results reported in [7]. These results show the chemical construction elucidated in Table 1. The MMD condition of the SIM and voltage was optimized to achieve the highest sensitivity (Table 2). As seen in Figure 2, the most intense transition was chosen to provide selective detection of phenols in the selective ion mode (SIM).

### 3.3. Method Validation

Calibration curves are obtained for six concentrations associated with triplicate injections. Good linearity was obtained for all analytes with correlation coefficients of *R*^2^ > 0.99 (Table 3). The LOD and LOQ were in the range of 0.5 *µ*g/mL–1 *µ*g/mL and 1 mg/kg–2 mg/kg, respectively. Average recoveries (Table 4) of target compounds at two fortified levels ranged from 83.2% to 97.5%. Repeatability represented by the RSD percentage was from 2.3% to 8.7%.

## 4. Contents of Phenols in *Dendrobium chrysotoxum*

The developed quantitative methods were applied to evaluate the level of phenols in *Dendrobium chrysotoxum.* The average contents of the four analytes, erianin, chrysotobibenzyl, confusarin, and moscatilin, were more than 200 mg/kg (Table 5). The content of bibenzyl was found to be the highest in *Dendrobium chrysotoxum*; the content of phenanthrene and confusarin was higher than that of flavone, but coumarin was not found in *Dendrobium chrysotoxum.* Among the target analytes, erianin was determined as the major component with the highest concentration of 425 mg/kg and can be considered as the typical chemical marker for quality evaluation and standardization of the botanical drug derived from *Dendrobium chrysotoxum.*

## Figures and Tables

**Table 1:** UPLC-MMD mass condition.

Compound	Class	Formula	Molecular weight	*m/z* (% relative abundant)
Gigantol GI	Bibenzyl	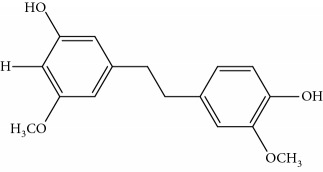	274	297 [M+Na]^+^ 100, 275 [M+H]^+^ 52,
Erianin ER	Bibenzyl	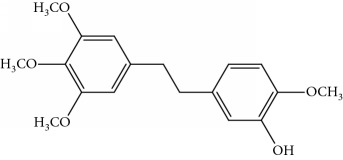	318	341 [M+Na]^+^ 100, 319 [M+H]^+^, 48
Chrysotobibenzyl CHB	Bibenzyl	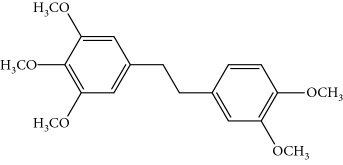	332	355 [M+Na]^+^ 100, 333 [M+H]^+^ 35
Tristin TR	Bibenzyl	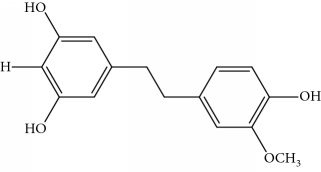	260	283 [M+Na]^+^ 100, 261 [M+H]^+^ 46
Moscatin MON	Phenanthrenes	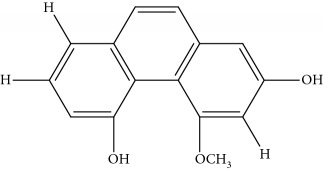	240	241 [M+H]^+^ 100, 213[M-28+H]^+^ 62
Confusarin COF	Phenanthrenes	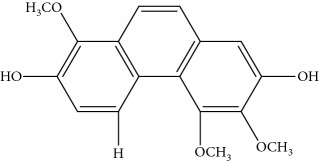	300	301 [M+H]^+^ 100, 269 [M-32+H]^+^ 48
Coumarin COM	Coumarin	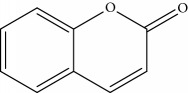	146	147 [M+H]^+^ 90
Naringenin NA	Flavone	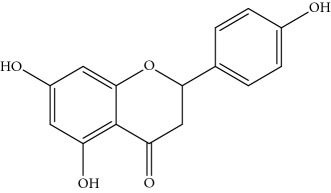	272	−271^a^ [M-H]^−^ 100
Apigenin AP	Flavone	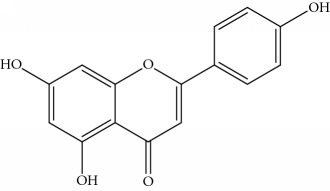	270	−269^a^ [M-H]^−^ 100
3,4-Dihydroxy-5,4′-dimethoxy bibenzyl DDB	Bibenzyl	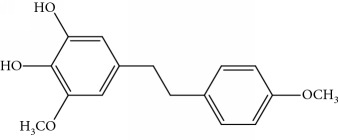	274	297 [M+Na]^+^ 100, 275 [M+H]^+^ 50
Moscatilin MOL	Bibenzyl	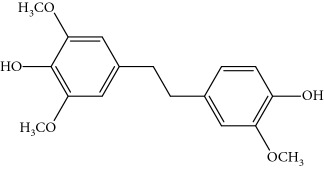	304	327 [M+Na]^+^ 100, 304 [M+H]^+^ 49
Chrysotoxin CHT	Bibenzyl	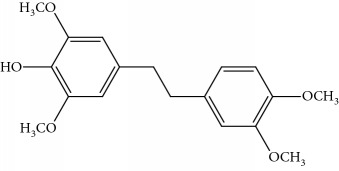	318	341 [M+Na]^+^ 83, 319 [M+H]^+^ 100

^a^Negative mode.

**Table 2:** UPLC-MMD parameters at the SIM mode.

Compound	*t*_R_(min)	SIM condition		Precursor ion (*m/z*)	Capillary volt (kV)	Cone volt (v)	Quantification with the corresponding standard
		Start time (min)	Stop time (min)				
GI	5.59	3	8	297	0.8	15	GI
ER	7.12	6	9.5	341	0.8	15	ER
CHB	1.85	0.5	3	355	0.8	15	CHB
TR	2.90	2	4	283	0.8	15	TR
MON	4.12	3	7	241	0.8	15	MON
COF	5.41	4	7	301	0.8	15	COF
COM	1.79	0.5	3	147	0.8	15	COM
NA	4.39	3	6	−271^a^	0.8	15	NA
AP	5.89	4.5	9	−269^a^	0.8	15	AP
DDB	5.62	3	8	297	0.8	15	GI
MOL	6.84	4	8	327	0.8	15	ER
CHT	6.97	6	9.5	341	0.8	15	ER

^a^Negative mode.

**Table 3:** Regression data, limit of detection (LOD), and limit of quantification (LOQ) of the proposed method.

	LOD (*µ*g/mL)	LOQ (mg/kg)	Calibration equation (*n*=5)	Determination coefficient, *R*^2^	Linear range (*µ*g/mL)
GI	1	2	*y* = 632964*x* − 95693	0.994	2–100
ER	1	2	*y* = 14667*x* + 3462	0.996	2–100
CHB	1	2	*y* = 718654*x* + 9443	0.994	2–100
TR	1	2	*y* = 474132*x* − 15725	0.996	2–100
MON	1	2	*y* = 874152*x* + 84434	0.998	2–100
COF	1	2	*y* = 364943*x* + 38924	0.997	2–100
COM	0.5	1	*y* = 14793*x* + 2938	0.995	1–100
NA	0.5	1	*y* = 261143*x* − 18631	0.993	1–100
AP	1	2	*y* = 914992*x* − 115674	0.999	2–100

**Table 4:** The recovery and RSD analyses of spiked samples at two concentrations (*n*=5).

	Sample	Spiked (50 mg/kg) (%)		Spiked (200 mg/kg) (%)	
		Recovery	RSD	Recovery	RSD
GI	12.7	95.8	4.3	83.2	2.3
ER	345	93.8	4.4	97.5	7.2
CHB	300	82.1	6.8	97.2	3.8
TR	15	91.8	6.1	91.5	6.7
MON	—	91.7	8.7	91.2	6.5
COF	250	93.6	6.3	89.4	6.2
COM	—	95.4	6.2	92.7	6.3
NA	6.26	90.8	5.2	88.5	5.4
AP	125	88.6	3.6	94.2	4.7
DDB	16.7	92.5	4.2	91.5	4.3
MOL	220	88.7	5.3	93.8	5.4
CHT	32	97.2	3.9	91.7	5.8

**Table 5:** The contents of *Dendrobium chrysotoxum* (mg/kg).

	Gigantol	Erianin	Chrysotobibenzyl	Tristin	Moscatin	Confusarin	Coumarin	Naringenin	Apigenin	3,4-Dihydroxy-5,4′-dimethoxy bibenzyl	Moscatilin	Chrysotoxin
1	12.7	345	300	15	—	250	—	6.26	8.5	16.7	220	32
2	8.9	520	324	—	35	280	—	8.93	5.9	18.6	187	21
3	—	450	253	12.3	—	267	—	15.4	12.3	22.4	248	26
4	23.4	387	264	—	24	189	—	8.4	15.7	25.8	265	27
Average	15	425	285	13.6	29.5	246.5	—	9.74	10.6	20.8	230	26.5

## Data Availability

The data used to support the findings of this study are included within the article.
